# Investigation of the Dermal Absorption and Irritation Potential of Sertaconazole Nitrate Anhydrous Gel

**DOI:** 10.3390/pharmaceutics8030021

**Published:** 2016-07-07

**Authors:** Mahima Manian, Kumpal Madrasi, Ayyappa Chaturvedula, Ajay K. Banga

**Affiliations:** 1 Department of Pharmaceutical Sciences, College of Pharmacy, Mercer University, Atlanta, GA 30341, USA; mahima.manian@live.mercer.edu; 2 Department of Pharmacy Practice, College of Pharmacy, Mercer University, Atlanta, GA 30341, USA; kumpal.madrasi@gmail.com (K.M.); ayyappach@gmail.com (A.C.); 3 Department of Pharmacotherapy, University of North Texas Health Science Center, Fort Worth, Texas 76107, USA

**Keywords:** topical, dermal absorption, diffusion model, skin irritation, sertaconazole nitrate

## Abstract

Effective topical therapy of cutaneous fungal diseases requires the delivery of the active agent to the target site in adequate concentrations to produce a pharmacological effect and inhibit the growth of the pathogen. In addition, it is important to determine the concentration of the drug in the skin in order to evaluate the subsequent efficacy and potential toxicity for topical formulations. For this purpose, an anhydrous gel containing sertaconazole nitrate as a model drug was formulated and the amount of the drug in the skin was determined by in vitro tape stripping. The apparent diffusivity and partition coefficients were then calculated by a mathematical model describing the dermal absorption as passive diffusion through a pseudo-homogenous membrane. The skin irritation potential of the formulation was also assessed by using the in vitro Epiderm™ model. An estimation of the dermal absorption parameters allowed us to evaluate drug transport across the stratum corneum following topical application. The estimated concentration for the formulation was found to be higher than the MIC_100_ at the target site which suggested its potential efficacy for treating fungal infections. The skin irritation test showed the formulation to be non-irritating in nature. Thus, in vitro techniques can be used for laying the groundwork in developing efficient and non-toxic topical products.

## 1. Introduction

Cutaneous fungal infections are not only widespread, but are responsible for many diseases of the skin and mucus membrane [1,2,3]. Up to 70% of the population worldwide are most commonly affected by a dermatophyte infection, usually tinea pedis [2]. In addition, 30%–40% of the population may be affected by pityriasis versicolor which, if left untreated, can progress to a more severe chronic condition called seborrheic dermatitis [1]. Over the past few decades, the HIV pandemic and the increasing use of immunosuppressive agents for treating cancer, autoimmune diseases and other medical conditions have resulted in an increase in the incidence and severity of fungal infections [3,4]. A number of azole antifungals are frequently used as a first line treatment for these superficial fungal infections. Sertaconazole nitrate (SN) is one such agent which was approved by the US FDA for treating tinea pedis in 2003. Chemically, SN is unique from other azole antifungals as it contains a benzothiophene ring which mimics tryptophan and increases the drug’s ability to form pores in the fungal cell membrane. The mechanism of action involves the inhibition of ergosterol synthesis resulting in increased cell wall permeability or leakage of cellular constituents which may ultimately result in cell death. In addition, the lipophilic benzothiophene ring aids in enhanced cutaneous retention [5] without increasing systemic absorption. The drug has both fungistatic and fungicidal activity against a variety of micro-organisms belonging to the *Dermatophytes* and *Candida* species [4,6,7,8]. The potency of sertaconazole to inhibit strains of *Malassezia* has also been reported [9,10]. All these studies demonstrate the broad spectrum antifungal activity of SN against a variety of fungal pathogens.

Currently, various topical formulations are available on the market for treating fungal diseases. The efficiency of topical antifungal therapy depends on the delivery of the active agent to the target site in adequate concentrations in order to exert its activity. An anhydrous vehicle is generally preferred as topical vehicle for azole antifungals [11] since the higher solubility of the drug in the vehicle may result in higher thermodynamic activity and, thus, greater partitioning into the skin. In this case, information about the dermatopharmacokinetic (DPK) profile of the topical formulation would help to assess the local bioavailability of the drug as well as provide a rationale for therapy. Tape stripping is one such technique which has been used to evaluate the bioavailability and bioequivalence of topical products [12,13]. This relatively noninvasive method has been used extensively to study the barrier function of skin [14,15] as well as to estimate the diffusivity and partition coefficients from in vivo dermal exposures [16,17]. These dermal absorption parameters can be used to quantify drug uptake in the SC and, thus, the absorption rate following topical application.

The objective of our research was to investigate the in vitro topical absorption of the model antifungal drug (SN) by using an anhydrous gel formulation. The topical absorption of the formulation was evaluated by in vitro tape stripping using both unsteady-state and steady-state experiments, similar to the methods reported in literature [18]. In addition to the bioavailability, it is also important to consider factors such as skin irritation and sensitization which may affect the subsequent safety and effectiveness of the product. Hence, the irritation potential of the formulation was evaluated by the in vitro Epiderm™ tissue model which has been validated by the European Centre for the Validation of Alternative Methods (ECVAM) and has been used since 2009 to assess skin irritancy [19].

## 2. Materials and Methods

### 2.1. Materials

SN was purchased from 2A Pharmachem (Lisle, IL, USA). PEG 400 and propylene glycol were provided by BASF (Tarrytown, NY, USA). Klucel^®^ (hydroxypropylcellulose) was provided by Ashland Inc. (Bridgewater, NJ, USA) while isopropyl myristate was from Croda Inc. (Edison, NJ, USA). Ascorbic acid and menthol were purchased from Sigma Aldrich (St. Louis, MO, USA) while glycerin was from Fisher Scientific (Waltham, MA, USA). Anhydrous ethanol (200 proof) was purchased from Acros Organic (Morris Plains, NJ, USA). All other reagents used were of high purity or HPLC grade.

### 2.2. Formulation of Anhydrous Gel

The gel was prepared in a glass jar at room temperature by slowly dispersing the gelling agent (Klucel^®^) in a mixture of glycerin, propylene glycol and anhydrous ethanol. The dispersion was continually stirred using a Teflon™-coated magnetic stir bar until a visually homogenous gel was formed. The 2% SN was gradually dissolved in polyethylene glycol (PEG) 400. Finally, isopropyl myristate, ascorbic acid, and menthol were added and the mixture was stirred continuously till a clear gel was obtained. Throughout the procedure, the jar was covered with Parafilm^®^ in order to prevent evaporation of ethanol. Prior to the formulation of the anhydrous gel, different vehicles were tested in order to determine the optimum ratio of the formulation for further permeation studies. The compositions of the formulations are shown in Table 1. The formulation was then optimized and the final composition of the anhydrous gel used is shown in Table 2.

### 2.3. In Vitro Permeation

Freshly excised porcine ear was obtained from a local abattoir no more than a few hours post-mortem and without any prior sanitization. The outer region of the skin was separated from the subcutaneous fat and stored at −80 °C until further use. Diffusion studies were performed using vertical Franz diffusion cells (Permegear, Hellertown, PA, USA). The thawed skin was clamped between the donor and receiver compartments of the diffusion cells. The temperature of the water bath was maintained at 37 °C in order to ensure a skin surface temperature of 32 °C. The receiver compartment contained PEG 400:1X PBS (phosphate buffered saline, pH 7.4) (60:40 *v*/*v*) which ensured sufficient solubility of the drug in order to maintain sink conditions. For the preliminary study, an infinite dose of the drug in different vehicles was applied on porcine skin. Once the formulation was optimized, a dose of 10 mg/cm^2^ of the anhydrous formulation was applied on porcine skin by using a positive displacement pipette. A minimum of four replicates was used for the entire study.

### 2.4. Tape Stripping

Following the diffusion studies, tape stripping was performed in vitro to quantify the amount of drug in the SC. In case of the anhydrous gel, the study was performed both for 5 min and for 24 h to establish an unsteady-state and steady-state profile, respectively. Based on the methods reported in [18], the unsteady-state experiment was performed when 0.03 ≤ *t*_exp_/*t*_lag_ ≤ 0.3 (where *t*_exp_ is duration of the exposure and *t*_lag_ is the lag time) in order to reduce large variations in estimating the lag time. The steady-state experiment was performed when *t*_exp_ > 1.7 *t*_lag_. Up to 20 pre-weighed adhesive tapes (3-M Transpore tape, USA) were cut into 2 × 2 cm square pieces, pressed onto the treated skin area and removed sequentially. Each tape strip was then weighed again and the mass of SC removed was determined. The tape stripping procedure was performed rapidly (less than 5 min) for both sets of experiments. The first tape strip was discarded due to potential contamination of residual drug on the skin surface. The remaining tapes were extracted individually for drug quantification by HPLC.

### 2.5. Skin Extraction

After tape stripping, the remaining stripped skin was minced finely and 1 mL of suitable extraction solvent (methanol) was added. After shaking for a suitable time period of 4 h to ensure adequate extraction of the drug from the tapes and skin, the samples were centrifuged, the supernatant was filtered and analyzed by HPLC.

### 2.6. Dermal Absorption Parameters Estimation

Data recorded in terms of mass of SC and mass of SN on tape strips were used to calculate concentration of SN (*C_n_*) in the tape strip using Equation (1) of [18]:

(1) $$C_{n}=m_{n}\rho_{sc}/m_{sc,n}$$

where *m_n_* is the mass of SN in the *n*^th^ tape strip, ρ*_sc_* is the density of the SC and *m_sc,n_* is the mass of the SC in the *n*^th^ layer. The depth of the SC from which the tape strip was drawn (*x_n_*) was calculated according to Equation (2) of [18]:

(2) $$x_{n}=\frac{1}{A\rho_{sc}}\left\{\frac{m_{sc,n}}{2}+\sum_{i=1}^{n-1}m_{sc,i}\right\}$$

where *A* is the area being tape stripped and *x_n_* is at the center of *m_sc,n_*. For this purpose, the density of the SC was assumed to be 1 g/cm^3^ [20] and the area of tape stripping was calculated to be 4 cm^2^. It was assumed that the process of tape stripping removed the entire SC. The thickness of the SC (*L_sc_*) was determined by Equation (3) of [18]

(3) $$L_{sc}=\frac{M_{sc}}{A\rho_{sc}}$$

where *M_sc_* is the sum of the entire SC obtained through tape stripping. It was assumed that dermal absorption occurs passively through a pseudo-homogenous membrane. Since SN is a hydrophobic compound and previous work characterizing skin to be a homogenous membrane for lipids has been published [21], this assumption was considered appropriate. These were then used to fit Equation (4) of [18] using the curve-fit toolbox of Matlab R2010a (Natick, MA, USA) to derive estimates on the partition coefficient *K*_sc/v_ between the SC and the vehicle and the lag time *t_lag_* for chemical to penetrate the SC under necessary assumptions.

(4) $$\frac{C_{n}}{K_{sc/v}C_{V}^{0}}=1-\frac{x}{L_{sc}}-\frac{2}{\pi }\sum_{n=1}^{\infty }\frac{1}{n}\exp\left(\frac{n^{2}\pi^{2}t_{\exp}}{6t_{\text{lag}}}\right)\sin\left(\frac{n\pi x}{L_{sc}}\right)$$

where $C_{V}^{0}$ is the initial concentration of the vehicle and *t*_exp_ is the time for which the drug exposure is allowed. The *t*_lag_ is defined as $L_{sc}^{2}/6D_{sc}$ where *D_sc_* is the effective diffusion coefficient in the SC. The parameter space used for identification of both the partition coefficient and the lag time ranged from zero to infinity. To ensure that Equation (4) remains valid, partition coefficient *K_sc_*_/*v*_ and the lag time *t*_lag_ were estimated after an exposure of 5 min. The density of the gel was assumed to be 10^6^ g/m^3^ and since SN concentration is 2% of the formulation, the initial concentration of the vehicle $C_{V}^{0}$ was considered as 20 × 10^3^ g/m^3^.

### 2.7. Diffusion Model

**Figure 1:**
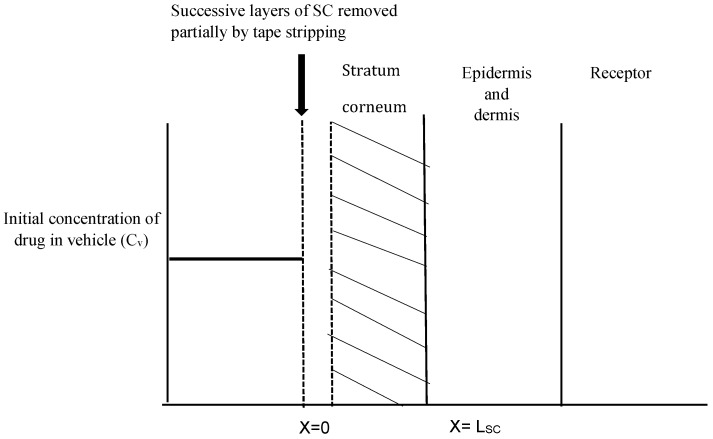
Schematic representation of the concentration-depth profile during the tape stripping experiment. Shaded region (i.e., stratum corneum) was stripped while drug concentration left in remaining stripped skin was recorded at end of experiment.

As seen in Figure 1, the one-layered diffusion model assumes that the SC is the rate-limiting barrier for topical absorption. With the removal of each tape strip, the outside edge of the barrier begins to move inward until no more SC is present. The vertical axis represents the concentration of the drug while the horizontal axis indicates the depth of the SC. The striped area represents the amount of penetrant in unit area of the skin.

The parameter estimates obtained were used to solve partial differential equations using the Matlab pdepe function to generate concentration time profiles. A 2D rectangular geometry of the same thickness as the SC was used to perform the simulations. Since SC is the primary site of action for anti-fungal drugs, the one layered diffusion model was used to simulate concentrations in the stratum corneum. The following equations as before [20] were used to simulate dermal absorption:

(5a) $$\frac{\partial C_{sc}}{\partial t}=D_{sc}\frac{\partial^{2}C_{sc}}{\partial x^{2}}$$

(5b) $$C_{sc}\int_{x=0}=K_{sc}\times C_{V}^{0}$$

(5c) $$C_{sc}\int_{x=L_{sc}}=0$$

(5d) *C*_sc_ = 0, for *t* = 0; 0 ≤ x ≤ *L*_sc_

Simulations of stratum corneum concentrations were conducted for 5 min and the concentration time profiles derived thereafter were used to examine the efficiency of SN in treating fungal infections for an exposure of 5 min. An MIC_100_ value of 2.695 µg/mL was used for yeasts [7,22] and an MIC_100_ value of 52 µg/mL was used for the fungal species of *M. furfur* [9].

### 2.8. Skin Irritation Testing

The anhydrous gel formulation was tested for skin irritation using the 3D tissue culture Epiderm™ model (MatTek Corporation, Ashland, MA, USA). As per the protocol, the tissues were pre-incubated to overcome shipping stress and then exposed to different treatments. The positive control (PC) was 5% SDS solution while negative control (NC) was sterile Dulbecco’s Phosphate Buffer Saline (DPBS). Tissues were exposed to 100 µL of the PC and the NC and 30 µL of the gel formulation for 1 h. Post-treatment, the tissues were incubated for 48 h. MTT assay was then carried out to determine the cell viability. The formulation was then classified as irritant/non-irritant based on the criteria in the given protocol.

### 2.9. HPLC Analysis

Quantification of SN was done using a validated method developed in our laboratory. The method was validated in terms of accuracy, precision and linearity and was found to be sensitive and specific for detection of SN. The analysis was carried out on an Alliance HPLC Water 2795 Separation Module consisting of an autosampler, a pump and a UV-diode array detector, using a Luna C18(2), 5 µm column (250 × 4.6 mm) (Phenomenex, Torrance, CA, USA). The column temperature was set at 40 °C and injection volume was 20 µL. The mobile phase consisted of 10 mM sodium phosphate buffer (pH, 3.0): acetonitrile (40:60 *v*/*v*). The flow rate was 1 mL/min and the detection wavelength was set to 225 nm.

## 3. Results

### 3.1. Localization of SN in Skin

**Figure 2:**
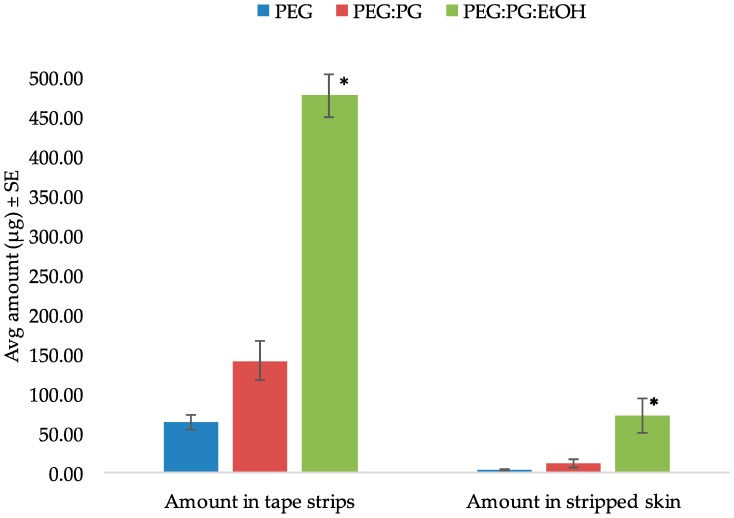
Amount of SN in tape strips and stripped skin using different vehicles (* indicates statistically significant compared to PEG and PEG:PG (*p* < 0.05)).

**Figure 3:**
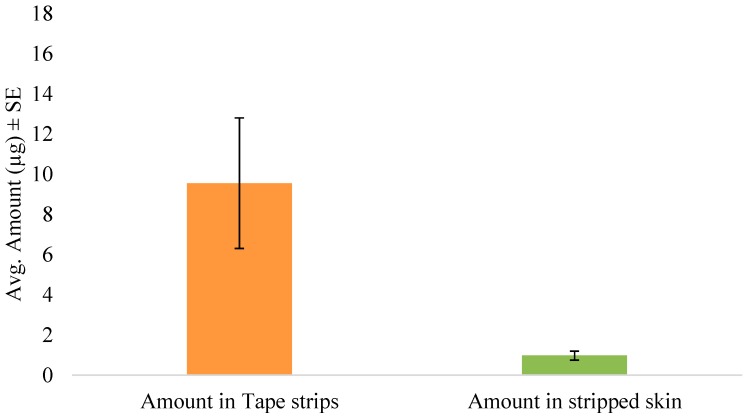
Amount of SN in tape strips and stripped skin following unsteady-state conditions (5 min).

**Figure 4:**
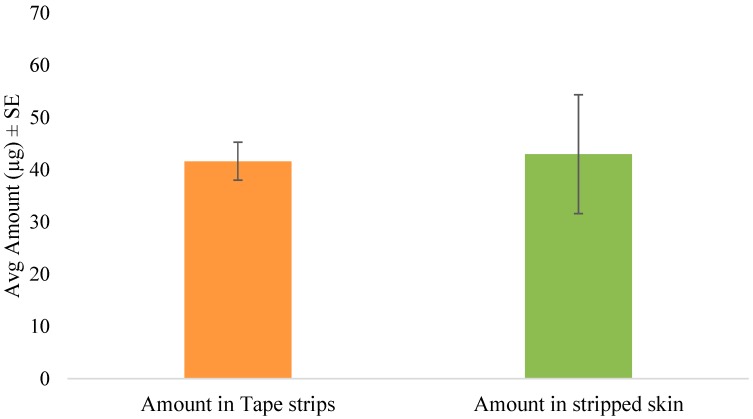
Amount of SN in tape strips and stripped skin following steady-state conditions (24 h).

The tape stripping method was used to determine the amount of SN in the skin following in vitro permeation. In the preliminary diffusion study, the inclusion of anhydrous ethanol significantly enhanced the skin retention of the drug as seen in Figure 2. Hence, the formulation was then optimized and used for further studies in the form of an anhydrous gel. After 5 min of formulation application of the gel, the skin was tape stripped immediately to simulate unsteady-state conditions. The amount of drug in the tape strips and stripped skin are shown in Figure 3. Thus, after immediate administration of the formulation, the drug had already distributed in the skin. Similarly, tape stripping was performed after 24 h to represent steady-state conditions and the results are shown in Figure 4.

**Figure 5:**
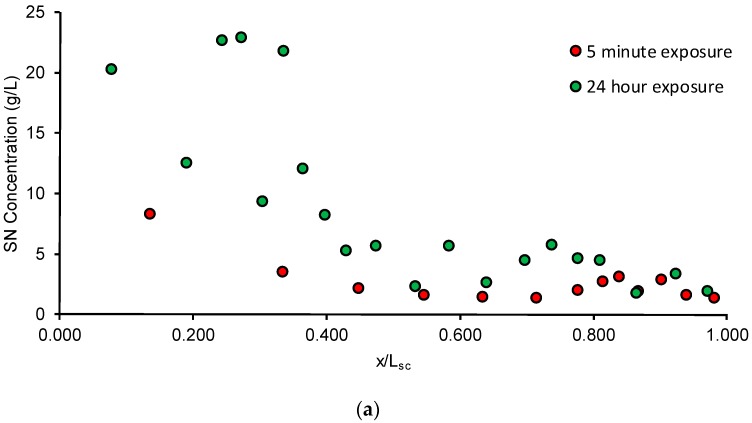

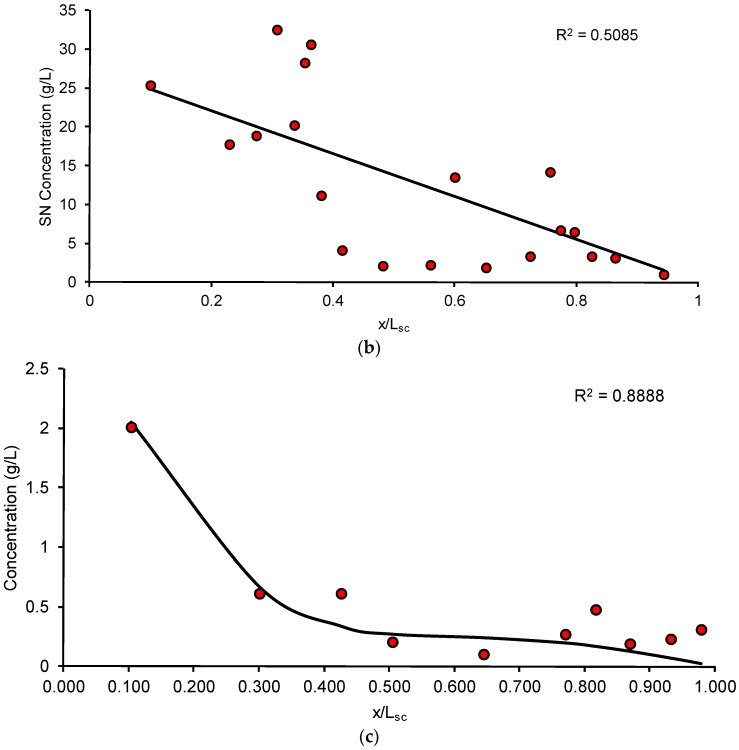
(**a**) Average SN concentration-depth profile following application of the formulation for 5 min and 24 h; (**b**) Sample fit for application of formulation for 24 h: markers are observed data and solid line is fit; (**c**) Sample fit for application of formulation for 5 min: markers are observed data and solid line is fit.

The SC concentration depth profile obtained after tape stripping is shown in Figure 5a. During the tape stripping, with each successive tape strip, a gradient from the outside to the inside was established which means that the drug penetrated throughout the layers of the SC. The amount of drug that penetrated the SC increased from 8.2% initially at the end of 5 min to 66.1% by the end of 24 h.

### 3.2. Parameter Estimation

As seen in Table 3, the thickness of the SC estimated using Equation (3) was found to be close to the physiologically reported SC thickness of the porcine ear skin [23]. The SN diffusivity and partition coefficient were calculated for an exposure time (*T*_exp_) of 5 min and 24 h. However, due to the poor quality of fits for the parameters estimated for the 24 h exposure, the parameters estimated from the 5 min exposure were used for the diffusion simulations. Comparison of the fits is done in Figure 5b, c and the parameters estimated are mentioned in Table 3.

### 3.3. Diffusion Model

**Figure 6:**
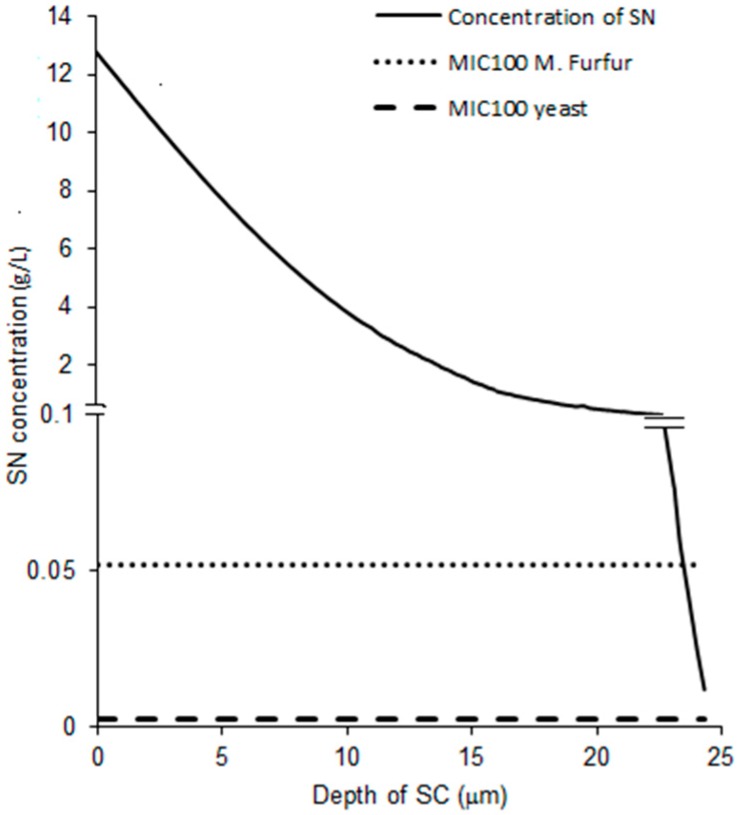
Simulated SN concentration across the SC plotted along with MIC_100_ of *M. furfur* and yeast.

Since very low amounts of the drug were found in the stripped skin for the experiment with the 5 min exposure, the single layer diffusion model, as considered neglecting diffusion to the dermis, should be a good, reasonable approximation for this period of exposure. The simulated concentration-depth profile following application of the formulation and tape stripping is shown in Figure 6. The concentration-depth profile obtained was compared with the minimum inhibitory concentration (MIC_100_) of fungal pathogens of interest, namely *M. furfur* and yeast. The resultant profile showed that the concentration of the drug stayed above the MIC_100_ value for yeast throughout the depth of the stratum corneum and above the MIC_100_ for *M. furfur* for almost the entire depth of the stratum corneum. This suggests that effective concentrations of sertaconazole may be achieved at the target site in patients following application of the formulation, thus indicating that the formulation is quite effective in treating fungal infections even after a brief exposure of 5 min.

### 3.4. Skin Irritation Test

**Figure 7:**
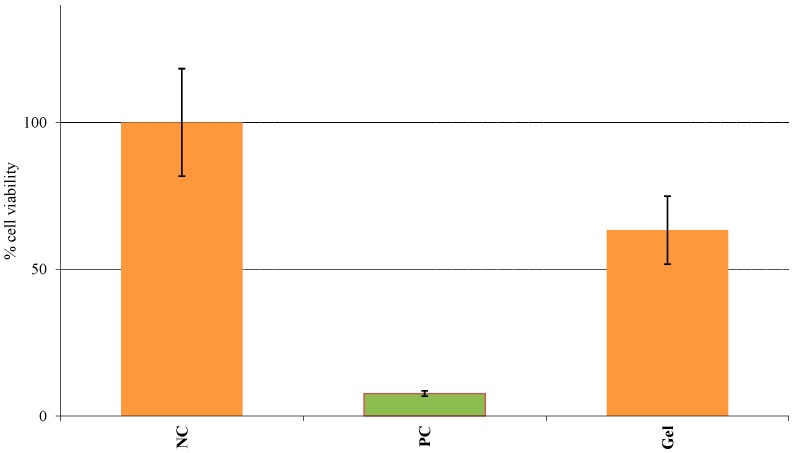
Skin irritation testing showed anhydrous gel to be non-irritant.

The relative percent viability of the Epiderm™ tissues treated with PC, NC and the anhydrous gel are shown in Figure 7. Based on the given protocol, any substance is considered as an irritant if the percentage of cell viability is less than 50% similar to the PC. On performing the MTT assay, the cell viability for the NC was considered to be 100% while the PC and the gel showed cell viability of 7.7% and 63.4% ± 11.62%, respectively.

## 4. Discussion

Effective topical therapy requires sufficient amounts of the drug in the skin in order to produce maximal pharmacological activity. The vehicle used can also have a significant effect on the permeation of the active ingredient. Ethanol has already been established as an effective penetration enhancer for lipophilic drugs where the mechanism of action may involve an increased solubility of the drug, and an increase in the thermodynamic activity of the drug due to evaporation of the ethanol or solubilization of the skin lipid [24,25]. Initial diffusion studies performed with different vehicles showed that the inclusion of anhydrous ethanol in the formulation significantly enhanced the distribution of the drug in skin. Hence, in order to enhance the cutaneous penetration of SN, which is highly lipophilic, an anhydrous gel containing ethanol was formulated. As shown in Table 2, the optimized formulation contained propylene glycol as a co-solvent, glycerin and PEG 400 as humectants, IPM as a lipophilic penetration enhancer and menthol for its antipruritic properties in an anhydrous vehicle. Also, no drug crystallization was observed in the optimized formulation which remained clear and transparent, indicating the stability of the drug in the anhydrous formulation. An anhydrous vehicle has the advantage of being well tolerated and acceptable without leaving any residue. Other adverse effects associated with the use of corticosteroids in treating certain infections or the use of greasy vehicles such as creams and ointments are also eliminated. Clinical studies using a commercially available anhydrous gel containing 2% ketoconazole have already shown the superior efficacy of this vehicle for the treatment of certain fungal diseases [11,26]. Hence, the anhydrous gel containing SN was formulated as it may show better effectiveness in treating cutaneous fungal infections due to all these advantages.

As per Guidance Document 2 established by the Organization for Economic Co-operation and Development [27], a significant number of studies substantiate direct comparisons of in vitro and in vivo methods. In vitro methods provide valuable data for the assessment of percutaneous absorption in humans. A good in vitro–in vivo correlation was observed when the bioavailability of the test and reference products were compared using excised human skin [28]. Recent clinical trials have demonstrated the superior efficacy of SN in treating tinea pedis and other fungal infections [29,30,31,32]. Hence, one of the main objectives of our study was to use SN as a model drug in order to demonstrate the rate and extent of in vitro drug absorption in skin and predict its local bioavailability. Establishing a dermal absorption profile helps in optimizing the formulation and improving the therapeutic efficacy of the product. Basically, the main objective is to maximize the drug concentration at the site of action (SC surface for antifungals) with minimal systemic uptake [33]. Previous in vivo studies done using 2% SN cream have showed a similar trend with undeterminable/minimum systemic bioavailability [34]. Our study also showed minimal amounts in the receptor (data not shown), similar to the in vivo situation. This outcome is reasonable since the study aims to show the absorption of the SN formulation at the target site rather than its systemic availability.

The DPK method or skin stripping has been used extensively to study the penetration of topically applied substances. Over the past decade, DPK has been regarded as a promising technique in the determination of the bioavailability and bioequivalence of topical products [35,36,37]. The typical procedure involves sequential removal of the SC by placing an adhesive tape onto the skin surface, applying pressure, and then removal by a sharp upward movement. This relatively noninvasive technique is useful for determining the rate and extent of drug absorption in the skin, especially in the case of drugs whose target is the SC, such as antifungals [38], keratolytics [39] and sunscreen agents [40]. Once the drug has been recovered and quantified from the extracted tape strips, the total amount in the SC can be determined. In this study, we used porcine ear skin to perform in vitro tape stripping as it has been established as a suitable model for simulating human skin [41,42]. The gravimetric approach was then used to quantify the amount of SC removed. Although the skin is a multilayered membrane, the SC and the underlying layers such as the viable epidermis are the main barriers to topical absorption. The one-layered diffusion model has been used previously [18,43] to predict skin concentrations following topical application. In the case of antifungals such as SN, the SC is the principal barrier and hence all the equations used in this study are based on the assumption that the SC is the rate-limiting barrier for dermal absorption. Hence, the one-layered diffusion model approach (Figure 1) was used in the parameter estimations. The clinical efficacy of the antifungal agent may be determined partly by the levels of the drug in the stratum corneum and also by the persistence of the drug at the target site for a suitable period which is enough to eradicate fungal growth [44]. Following tape stripping, as seen in Figure 3 and Figure 4, the amount of drug in the SC and deeper layers was determined from experiments with two different times of exposure. By the end of the 24 h exposure to the formulation, approximately 66% of the applied dose had penetrated the skin. Even though this may seem slightly low, about 8% of the dose was already present in the skin after 5 min of application.

As seen in Figure 5a, the calculated SC concentration normalized depth profile was determined as a function of time following application of the formulation. After 24 h, although the time required to tape strip was lesser than the lag time, it is possible that some drug may have still diffused during the tape stripping process, resulting in a non-linear profile (Figure 5b). In comparison, the profile after application for 5 min shows an inverse linear relationship between the drug concentration and SC depth (Figure 5c) in spite of the fact that drug in only 10 tape strips could be quantified potentially due to sensitivity constraints of the analytical method. However, studies have shown that for determining the SC thickness and for the interpretation of the concentration-depth profile, complete ablation of the barrier is unnecessary for determining the required information [45]. The results of the parameter estimation following the tape stripping procedure are summarized in Table 3. The SC thickness of porcine skin was found to be approximately 23.7 ± 4.0 µm and 24.4 ± 3.3 µm for steady-state and unsteady-state conditions, respectively, which are close to the reported values in literature [23]. This indicates that after 5 min, by our estimation, the SC may have been removed almost completely. The lag times estimated for the 5 min and 24 h exposure experiments were 0.43 ± 0.09 h and 0.41 ± 0.23 h, respectively. Thus, for the 5 min exposure, the ratio of *t*_exp_/*t*_lag_ is approximately 0.19, while for the 24 h exposure, the ratio of *t*_exp_/*t*_lag_ is approximately 58.2. As mentioned in Section 2.4, our unsteady-state experiment was thus conducted in the range of 0.03 ≤ *t*_exp_/*t*_lag_ ≤ 0.3 while our steady-state experiment was conducted in the range of *t*_exp_/*t*_lag_ > 1.7, hence satisfying the recommendations put forward by [18]. The partition coefficient and lag times were then estimated for 5 min and 24 h. It has been reported that in the case of a volatile vehicle which begins to evaporate from the moment of application, drug uptake was the same for both a short duration of exposure as well as after the chemical was allowed to stay on the skin for a more extended duration [46]. Hence, the estimated parameters for the 5 min exposure represent a reasonable estimate of drug uptake into the skin. The partition coefficient *K_sc/v_* determines the affinity of the drug for the SC compared to the applied formulation. The diffusivity coefficient was estimated by the relationship *D_sc_* = *L_sc_*^2^/ (6 × *t*_lag_). Thus, the drug concentration–SC depth profile can be used to estimate both thermodynamic and kinetic parameters which are useful to characterize drug uptake in the skin following application of the formulation.

Due to the better fit of data from the 5 min exposure, the profile obtained from the 5 min exposure data was chosen for further simulation. The MIC_100_ represents the lowest concentration of the drug that can cause total inhibition of fungal growth The simulated concentration-depth profile of SN (Figure 6) proved to be higher than the literature-derived MIC_100_ of yeasts for the entire thickness of the stratum corneum and higher than the literature-derived MIC_100_ of *M. furfur* for almost the entire thickness of the stratum corneum. Thus, the exposure levels attained by the drug may be sufficient to treat superficial fungal infections caused by these pathogens once steady-state is achieved.

One of the major limitations in the model fit after steady-state data is the violation of the sink boundary condition at the end of the stratum corneum, as we see quantifiable amounts of drug detected beyond the stratum corneum layers, which was considered to be an in vitro artifact induced by lack of sink conditions in the experiments. Although it is a common assumption to assume sink conditions in vivo, we have observed subdermal layers having significant drug levels in microdialysis experiments and thus this assumption is not entirely valid [47]. In this work, also, the boundary condition is reasonable as the drug levels were, on average, five folds less in skin layers below SC (Figure 3) compared to the SC. This could be possibly the reason for the better fit to the 5 min exposure data as the amount in the deeper layers below the SC was only 0.97 ± 0.45 µg compared to 42.95 ± 22.78 µg by the end of 24 h. We hypothesize that this violation could potentially be a function of drug lipophilicity and further exploration needs to be conducted to generalize the results.

In addition to the therapeutic outcome, an excellent safety profile is an integral part of any topical therapy. Skin irritation can have a significant influence on therapeutic effectiveness and may result in adverse reactions. SN has already been well established as safe and non-toxic in the treatment of fungal infections [48,49]. In some cases, the cause of skin irritation may be the vehicle and not the drug itself. Since the gel contained a large percentage of ethanol, the final step of our study involved evaluating the irritation potential of the formulation. Conventional irritation testing involves the use of the Draize test which may cause severe pain and suffering in animals, depending on the irritation produced by the specific agent. This method was subsequently banned by ECVAM and has been replaced by validated in vitro human epidermal models. The Epiderm™ model consists of three-dimensional human epidermal keratinocytes comprised of an intact basal layer along the SC and viable epidermis. As per the given protocol, any formulation showing less than 50% cell viability following the MTT assay can be considered an irritant. As seen in Figure 7, since the percentage cell viability of the anhydrous gel was greater than 50% similar to the NC, the formulation can be classified as non-toxic and non-irritant [50].

## 5. Conclusions

In this study, we evaluated the dermal absorption of SN anhydrous gel by in vitro tape stripping in order to understand the local bioavailability in skin. A mathematical model was then established for estimating the partition and diffusion coefficients. These dermal absorption parameters were used to predict the topical absorption of the drug up to 5 min. The estimated concentration time profile was found to predict a greater concentration than the MIC_100_ for *M. furfur* and yeast for almost the entire depth of the stratum corneum. This suggests that the formulation may reach the target site in adequate concentrations to effectively treat fungal infections. In order to evaluate the toxicity of the formulation, the Epiderm™ skin irritation test was then performed which categorized the anhydrous gel formulation as non-irritating. Thus, these in vitro techniques are extremely useful surrogate tools in the development of topical formulations with improved therapeutic efficacy and safety.

## Figures and Tables

**Table 1:** Composition of SN (2% *w*/*w*) in different vehicles.

Components	Formula A (%)	Formula B (%)	Formula C (%)
Sertaconazole Nitrate (SN)	2	2	2
Propylene glycol	98	49	25
PEG 400	-	49	25
Ethanol, anhydrous	-	-	48

**Table 2:** Anhydrous gel composition.

Components	Formula (%)
Sertaconazole Nitrate (SN)	2
Propylene glycol	20
Klucel	2
Glycerin	15
PEG 400	20
IPM	2
Menthol	1
Ascorbic acid	0.2
Ethanol, anhydrous	37.8

**Table 3:** Details of TS experiment and parameter estimation.

Experimental Details		Estimated Parameters		
	*T*_exp_	*L_sc_* (µm)	*K_sc_*_/*v*_	*D_sc_* (m^2^/s)
5 min exposure	5 min	24.4 ± 3.3	0.64 ± 0.51	(6.47 ± 1.56) × 10^−14^
24 h exposure	24 h	23.7 ± 3.95	0.98 ± 0.37	(8.88 ± 6.4) × 10^−14^

*L_sc_*: Thickness of SC; *K_sc_*_/*v*_: Partition coefficient of drug between SC and vehicle; *D_sc_*: Diffusion coefficient in the SC.
